# Examining the Mental Health Presentations of Treatment-Seeking Transgender and Gender Nonconforming (TGNC) Youth

**DOI:** 10.1007/s10578-021-01289-1

**Published:** 2021-12-03

**Authors:** Shannon L. Stewart, Jocelyn N. Van Dyke, Jeffrey W. Poss

**Affiliations:** 1 Faculty of Education, University of Western Ontario, 1137 Western Rd, London, ON N6G 1G7 Canada; 2 School of Public Health Sciences, University of Waterloo, 200 University Ave W, Waterloo, ON N2L 3G5 Canada

**Keywords:** TGNC, Transgender, Gender nonconforming, Adolescent mental health, interRAI

## Abstract

Recent research suggests that transgender and/or gender nonconforming (TGNC) youth present with heightened levels of mental health problems compared to peers. This study seeks to examine the mental health needs of a large sample of treatment-seeking TGNC youth by comparing them to cisgender males and females. Participants were 94,804 children and youth ages 4–18 years (*M* = 12.1, SD = 3.72) who completed the interRAI Child and Youth Mental Health Instrument (ChYMH) or Screener (ChYMH-S) at participating mental health agencies in the Ontario, Canada. Overall, the mental health presentations of TGNC youth were similar to cisgender females but at higher acuity levels. TGNC youth showed significantly higher levels of anxiety, depression, social disengagement, positive symptoms, risk of suicide/self-harm, and were more likely to report experiencing emotional abuse, past suicide attempts, and a less strong, supportive family relationship than cisgender females and males. Clinical implications of these findings are discussed.

## Introduction

Over the years, there has been an increased awareness for the mental health needs of transgender and/or gender nonconforming (TGNC) individuals. TGNC individuals, or those whose gender identity does not align with their birth-assigned sex, are an understudied population, especially in child and youth populations [1, 2]. TGNC children and youth (hereafter referred to as youth) are particularly vulnerable as they are at an increased risk of abuse both inside and outside the home [3–5] and are disproportionately exposed to a number of risk factors including bully victimization [1, 5–8], traumatic experiences [3], high-risk sexual behaviours [1, 9], and substance use [1, 3, 7]. Furthermore, this population experiences significantly lower levels of protective factors than their cisgender peers, including a lack of perceived social support [3] and lower levels of internal assets, family connectedness, student–teacher relationships, and feeling safe in the community [1].

In addition to this disproportionate exposure to psychological stressors early in life, there is a growing body of literature demonstrating mental health disparities in TGNC youth populations. Gender nonconformity in childhood before 11-years-old is a risk factor for lifetime probable post-traumatic stress disorder (PTSD) [4] and depressive symptoms [5] in early adulthood. Compared to their cisgender peers, TGNC youth report heightened levels of mental health symptomology including higher rates of psychological distress [10], attention deficit disorders between the ages of 3 and 9-years-old [11], self-harm [1, 10–13], anxiety [11, 13–15], and depression [1, 3, 6, 10, 11, 13, 14, 16].

Recent literature suggests that suicidality, whether considered or attempted, is also high in this population [1, 3, 6, 10, 11, 13, 16, 17]. A Canadian study found that 65% of transgender youth aged 14–18-years-old had seriously considered suicide in the past year, a rate that was much higher than their cisgender peers [10]. Furthermore, Minnesota high school students who identify as TGNC are over three times more likely to report suicidal ideation, and 31% reported an actual suicide attempt [1]. In a study of transgender high school students from New Zealand, approximately 20% had attempted suicide, and over 40% had depressive symptoms and had self-harmed in the past year [6]. Despite the various mental health disparities of TGNC youth explored in the emerging literature thus far, there is still a need for large-scale studies which provide a comprehensive examination of TGNC youth’s mental health needs.

### Current Study

The present study seeks to fill this gap in the literature by using a large Canadian sample of treatment-seeking TGNC youth. To provide a comprehensive understanding of the various mental health presentations of treatment-seeking TGNC youth, the present study examines demographic and mental health service trends from intake assessments at mental health agencies across Ontario, Canada. To the best of our knowledge, this is the first Canadian study to include a large, treatment-seeking sample of TGNC youth. Furthermore, this study adds to the current literature as it compares TGNC mental health presentations to cisgender males and females separately. Aligned with previously cited literature, we anticipate that gender will be highly related to youth mental health presentations, with TGNC individuals overall showing disproportionately higher mental health needs than both cisgender males and females. This research is critical in understanding the different mental health service needs of TGNC, and cisgender male and female youth in order to accurately inform care-planning and prevent subsequent negative sequalae.

## Methods

### Sample

Data was obtained from assessments of referred youth to mental health agencies in Ontario. As part of regular clinical practice, these individuals were assessed with either the Child and Youth Mental Health-Screener (ChYMH) or the Child and Youth Mental Health (ChYMH) full assessment, described below. Assessment responses, mostly binary or ordinal scale measures, were entered using secure on-line software which constrains the responses to the proper form and to be all complete prior to the assessment record being submitted. All assessments between November 2012 and August 2020 with valid responses for sex and gender were used, and if an individual was assessed more than once during this time the first assessment was used. The options of male (M), female (F), and other (O) were provided for both sex and gender. Those who endorsed the gender category of male and female are assumed to be cisgender for the purposes of this study, and those who endorsed the category of “other” are reflected as TGNC, as this is a broad term commonly used in the literature and best represents this group within the sample.

There were 78,646 individuals assessed with the ChYMH-S and 16,158 with the ChYMH full assessment, for a total of 94,804 observations from 71 service organizations.

### Measures

#### interRAI ChYMH

The interRAI Child and Youth Mental Health Assessment (ChYMH) is a comprehensive assessment which includes over 400 items divided into 22 sub-sections to evaluate and identify youth’s mental health needs, risks, and inform care-planning [18]. Trained assessors obtain information from multiple sources including information from the youth, their caregivers, teachers, and clinicians, as well as available medical and education records. The ChYMH has a variety of scales and algorithms embedded within the instrument to support clinicians in obtaining a data-driven picture of the youth’s strengths, needs, functioning, and areas of risk.

This ChYMH is part of the Child and Youth suite of interRAI assessment tools, in which the scales and algorithms demonstrate robust psychometric properties [e.g. 19, 20, 21, 22, 23, 24, 25, 26, 27]. More information regarding the interRAI assessment tools can be found on the interRAI website (www.interrai.org).

#### interRAI ChYMH Screener

The interRAI ChYMH Screener (ChYMH-S) is a brief, 99 item, initial screening assessment used for the purpose of assisting in decision-making related to triaging and the prioritization of services [28]. The ChYMH-S has been adapted from the ChYMH and uses semi-structed interviews to provide a snapshot of the various aspects of the child’s functioning and aids in determining if a more comprehensive assessment is needed.

#### Scales and Algorithms

Note that for the following scales and algorithms, the analysis employed 4 collapsed groupings for each scale, rather than treating the scale as a continuous measure. This aids in interpretation as well as helping to manage non-linearities across the scale range. The choice of cut-points is based on accepted standards that interRAI (see www.interrai.org) has made available to those using these data, to encourage uniformity and comparability across studies. Four groups are labelled low, moderate, high, and very high based on the performance of each scale as part of their respective derivations.

##### The Depressive Severity Index (DSI)

The DSI is a five-item scale embedded within several interRAI Child and Youth Instruments measuring depressive symptoms (i.e., sad or pained facial expression, made negative statements, self-deprecation, expressions of guilt/shame, and hopelessness). Each item is given a score from 0 to 3, and are coded as: 0 (“Not present”), 1 (“Present but not exhibited in last 3 days”), 2 (“Exhibited on 1–2 of last 3 days”), and 3 (“Exhibited daily in last 3 days”). The DSI was originally developed by Perlman and colleagues [29] and shows strong psychometric properties in youth populations [30].

##### Risk of Injury to Others (RIO) Algorithm

The RIO algorithm measures the risk of harm to others and is a psychometrically sound instrument in clinically-referred youth populations [24]. The algorithm uses nine items including violent ideation, threatened violence, violence to others, verbal abuse, socially inappropriate or disruptive behaviour, family overwhelmed, impulsivity, and physical abuse. It includes an empirically based decision tree designed to indicate risk levels, with higher risk levels indicating greater risk of injury to others. Levels of risk range from zero to six, with a cut-point of 3 + indicating severe risk of injury to others.

##### Risk of Suicide and Self-Harm in Kids (RiSsK) Algorithm

The RiSsK algorithm uses a decision tree composed of six items reflecting the risk of suicide and self-harm in youth. These items include attempt to kill, self-harm without intent to kill, considered self-injury, others concerned about self-injury, family overwhelmed, and any self-injurious behaviours. Levels of risk range from zero to six, with a cut-point of 2 + indicating risk of suicide and self-harm among clinically-referred youth populations. The RiSsK has strong psychometric properties which support its clinical utility [23].

##### Anxiety Scale

Frequency of anxiety symptoms are measured using the Anxiety Scale. This scale consists of six items (i.e., anxious complaints or concerns, unrealistic fears, obsessive thoughts, intrusive thoughts or flashbacks, episodes of panic, and nightmares). The items are scored on a scale from zero to four (i.e., 0 = Not present, 1 = Previously present but not exhibited in last 3 days, 2 = Exhibited on 1–2 of last 3 days, and 3 = Exhibited daily in last 3 days, 1–2 episodes, or 4 = Exhibited daily in last 3 days, 3 or more episodes or continuously), with higher total scores indicating higher levels of anxiety. Psychometric evaluations support the scale’s use in youth populations [22].

##### Externalizing Scale (Short)

The Externalizing Mental Health Scale—Short (CY-EXT-S) is a psychometrically sound measure of the frequency and severity of both proactive and reactive aggression, violence, and impulsivity. These constructs are assessed through seven items: stealing, bullying peers, impulsivity, verbal abuse, violence to others, violent ideation, intimidation of others, and threatened violence. Each score was dichotomized (0 not present; 1–4 present) and summed to produce a total score, with higher scores being evident of more severe externalizing behaviours [21].

##### Positive Symptoms Scale (PSS)

PSS is a four item scale which measures the presence of positive symptoms including hallucinations, command hallucinations, delusions, and abnormal thought process/form. The scores from each of these four items is summed to reveal final scores ranging from zero to twelve, with higher scores representing heightened levels of positive psychotic symptomology [19].

##### Hyperactive/Distraction Scale (HDS)

The frequency of hyperactivity and distractibility is assessed by the HDS. Each of the four items on the scale (i.e., impulsivity, ease of distraction, hyperactivity, and disorganization) is scored from zero to four, with zero indicating symptoms not present and four being symptoms exhibited daily is last three days, three or more episodes or continuously. The highest possible score is sixteen, indicating the highest levels of hyperactivity and distractibility [20].

##### Social Disengagement Scale (SDS)

Previously known as the Anhedonia Rating Scale, the SDS assesses the frequency of anhedonia and social disengagement through four items: lack of interest in social interaction, lack of motivation, anhedonia, and withdrawal from activities of interest. Each item is scored on a scale of zero (symptoms not present) to four (symptoms exhibited daily in the last three days, three or more episodes or continuously). Each item is summed to provide a maximum score of sixteen, with higher scores indicating more anhedonia [25].

### Procedure

As part of routine clinical practice, trained assessors such as nurses, psychologists, psychiatrists, social workers, children and youth workers, case managers, and speech language pathologists administered the ChYMH and ChYMH-S across 71 mental health service agencies in Ontario. At each agency, informed consent was obtained from all individual participants and/or guardians as part of standard of care. Data from the ChYMH and ChYMH-S was obtained through 60–90 min semi-structured interviews with the youth, their caregivers, teachers, and/or clinicians, as well as medical and education records. Each complete ChYMH and ChYMH-S assessment was given a case number and stored on a secure server with no identifying information such as names, full birthdays, and postal codes to ensure anonymity. Ongoing access to this server for research purposes has been approved by the University of Western Ontario Ethics Board (REB #106415) and all procedures were in accordance with the ethical standards of the institution. All analyses used in this study were conducted on SAS version 9.4 software (SAS Institute, Cary, NC, USA).

### Analysis

Descriptive summaries were created of the distributions of selected mental health measures by client gender: cisgender male, cisgender female, and TGNC. Since age differed significantly between genders, pair-wise tests of mental health measurements (cisgender female compared to cisgender male, TGNC compared to cisgender male, and TGNC compared to cisgender female), controlled for age as a continuous variable. Specifically, logistic regression was used, with age and gender as independent variables and the various descriptive items and scales as the dependent variable. Odds ratios are reported, with 95% confidence intervals, except for some descriptive characteristics (language, lives with caregiver, in school) where simple statistical significance of gender with 95% confidence is reported.

We chose not to apply a family-wise error rate adjustment to the group of findings (such as the Bonferroni correction). Such a correction is influenced by the size of the family of measures (more measures will result in a more conservative degree of correction), and we chose to opt for a larger set of measures, many of which are correlated. Correlation among a family of measures can result in an over-correction in a Bonferroni or related adjustment [31]. However, caution is advised regarding some reported significant findings where the reported odds ratios are wide and come close to overlapping one.

## Results

Table 1 shows the overall sample with selected demographic/descriptive measures. Cisgender males made up 50.4% of the sample, cisgender females 48.9% and TGNC gender 0.7%. Among those with a gender of TGNC, 68% were recorded as being biologically female, 13% biologically male, and 19% biologically neither male nor female. Compared to cisgender females, cisgender males were about 1.7 years younger and TGNC youth about 1.9 years older. English and primary language and living with a parent/primary caregiver did not differ by gender, after adjusting for age. While most of the sample were reported to be in school, cisgender females had higher likelihood, adjusting for age, compared to cisgender males (p < 0.0001).

**Table 1:** Sample description

	Gender							
	Cisgender male		Cisgender female		TGNC			All
	%	*N*	%	*N*	%	*N*	%	*N*
Sex (all)		47,805		46,333		666		94,804
Biological male	98.5	47,099	0.4	189	13.1	87	50.0	47,375
Biological female	1.4	689	99.6	46,129	68.5	456	49.9	47,274
Biological other	0.0	17	0.0	15	18.5	123	0.2	155
Age (*mean and standard deviation*)	*11.0*	*3.82*	*12.7*	*3.54*^a^	*14.6*	*2.21*^b,c^	*12.1*	*3.72*
0–7	21.8	10,406	11.1	5160	1.4	9	16.4	15,575
8–11	32.0	15,277	20.3	9392	5.7	38	26.1	24,707
12–15	31.1	14,858	43.6	20,193	57.7	384	37.4	35,435
16 and older	15.2	7264	25.0	11,588	35.3	235	20.1	19,087
Primary language is English	95.3	45,558	95.7	44,358	97.3	648	95.5	90,564
Lives with parent/primary caregiver	92.1	44,009	91.2	42,238	89.0	593	91.6	86,840
In school	96.0	45,764	96.7	44,698^d^	95.6	634	96.3	91,096

% and *N* indicate percent of gender and number of individuals, respectively

^a^Significant difference cisgender female compared to cisgender male

^b^Significant difference TGNC compared to cisgender male

^c^Significant difference TGNC compared to cisgender female

^d^Significant difference cisgender female compared to cisgender male, adjusting for age

Table 2 presents selected mental health measures and scales available in the assessment data. Prevalence/distribution as well as odds ratios, adjusting for age, of the three pair-wise gender comparisons are summarized.

**Table 2:** Mental health measures

	Gender						All		Odds ratios (95% CI), adjusting for age					
	Cisgender Male		Cisgender Female		TGNC				Cisgender Female vs Cisgender Male		TGNC vs Cisgender Male		TGNC vs Cisgender Female	
	%	*N*	%	*N*	%	*N*	%	*N*	OR	95% CI	OR	95% CI	OR	95% CI
Self-injurious attempt to kill self	5.6	2684	11.7	5415	24.5	163	8.7	8262	**1.62**	**1.54–1.71**	**2.96**	**2.46–3.56**	**1.82**	**1.52–2.19**
Strong supportive family relationship	88.7	42,385	83.9	38,882	73.6	490	86.3	81,757	**0.84**	**0.81–0.88**	**0.56**	**0.47–0.67**	**0.67**	**0.56–0.80**
Family report feeling overwhelmed	40.8	19,478	32.2	14,896	29.0	193	36.5	34,567	**0.77**	**0.75–0.79**	**0.76**	**0.64–0.90**	0.98	0.83–1.17
Victim in the last year														
Physical assault/abuse	4.1	1964	5.1	2352	6.6	44	4.6	4360	**1.10**	**1.03–1.17**	1.28	0.94–1.74	1.16	0.85–1.58
Sexual assault/abuse	0.7	318	4.3	1970	6.6	44	2.5	2332	**5.48**	**4.89–6.18**	**7.20**	**5.19–9.99**	1.31	0.96–1.79
Emotional abuse	8.3	3,984	14.1	6508	21.5	143	10.8	10,278	**1.54**	**1.48–1.61**	**2.20**	**1.82–2.65**	**1.43**	**1.18–1.72**
Bullying	22.7	10,845	23.5	10,908	25.4	169	23.1	21,922	**1.10**	**1.06–1.13**	**1.27**	**1.06–1.51**	1.16	0.97–1.38
Depressive symptom inventory (DSI)														
Low: 0	17.2	8224	11.7	5422	7.2	48	14.4	13,694	Ref		Ref		Ref	
Moderate: 1–3	34.5	16,488	30.2	13,994	18.3	122	32.3	30,604	**1.25**	**1.20–1.30**	1.17	0.84–1.64	0.94	0.67–1.32
High: 4–7	30.0	14,336	30.6	14,191	31.2	208	30.3	28,735	**1.35**	**1.29–1.41**	**1.95**	**1.42–2.67**	**1.45**	**1.05–1.98**
Very high: 8–15	18.3	8756	27.5	12,726	43.2	288	23.0	21,770	**1.77**	**1.70–1.86**	**3.54**	**2.60–4.82**	**2.00**	**1.47–2.72**
Risk of suicide/Self–Harm (RISsK)														
Low: 0	52.8	25,237	38.7	17,914	12.6	84	45.6	43,235	Ref		Ref		Ref	
Moderate: 1–2	28.2	13,460	26.4	12,245	26.1	174	27.3	25,879	**1.14**	**1.10–1.17**	**2.92**	**2.25–3.80**	**2.57**	**1.98–3.34**
High: 3–4	14.9	7112	25.5	11,811	41.4	276	20.3	19,199	**1.78**	**1.71–1.84**	**6.43**	**5.01–8.24**	**3.62**	**2.82–4.64**
Very high: 5–6	4.2	1988	9.4	4359	19.8	132	6.8	6479	**2.27**	**2.14–2.41**	**10.32**	**7.79–13.67**	**4.55**	**3.44–6.01**
Risk of injury to others (RIO)														
Low: 0	40.8	19,502	66.7	30,881	73.9	492	53.7	50,875	Ref		Ref		Ref	
Moderate: 1–2	23.1	11,060	15.1	6975	14.0	93	19.1	18,128	**0.46**	**0.45–0.48**	**0.49**	**0.39–0.561**	1.95	0.85–1.33
High: 3–4	27.1	12,932	14.8	6853	10.4	69	21.0	19,854	**0.42**	**0.40–0.43**	**0.39**	**0.30–0.50**	0.93	0.72–1.21
Very high: 5–6	9.0	4292	3.5	1610	1.8	12	6.2	5914	**0.30**	**0.29–0.32**	**0.23**	**0.13–0.41**	0.75	0.42–1.33
Anxiety scale														
Low: up to 2	46.9	22,314	35.2	16,276	23.0	153	41.0	38,743	Ref		Ref		Ref	
Moderate: 3–7	34.8	16,556	36.9	17,060	34.1	227	35.8	33,843	**1.38**	**1.34–1.42**	**1.90**	**1.55–2.34**	**1.38**	**1.12–1.69**
High: 8–12	12.8	6096	18.1	8368	25.0	166	15.5	14,630	**1.75**	**1.68–1.82**	**3.40**	**2.73–4.25**	**1.94**	**1.56–2.43**
Very high: 13–28	5.6	2657	9.9	4565	17.9	119	7.8	7341	**2.03**	**1.93–2.14**	**4.81**	**3.77–6.14**	**2.37**	**1.86–3.02**
Externalizing (Short Form)														
Low: up to 2	21.9	10,451	41.3	19,120	41.6	277	31.5	29,848	Ref		Ref		Ref	
Moderate: 3–7	28.5	13,604	32.0	14,814	36.5	243	30.2	28,661	**0.65**	**0.63–0.67**	**0.83**	**0.69–0.98**	**1.27**	**1.07–1.52**
High: 8–12	34.0	16,240	19.6	9065	17.1	114	26.8	25,419	**0.38**	**0.37–0.40**	**0.47**	**0.38–0.59**	1.24	0.99–1.55
Very high: 13–28	15.7	7509	7.2	3334	4.8	32	11.5	10,875	**0.30**	**0.29–0.32**	**0.29**	**0.20–0.42**	0.95	0.66–1.38
Positive symptoms scale														
Low: 0	89.8	42,924	88.4	40,940	72.4	482	89.0	84,346	Ref		Ref		Ref	
Moderate: 1–2	6.6	3166	7.6	3521	17.6	117	7.2	6804	1.00	0.95–1.05	**2.44**	**1.98–3.00**	**2.43**	**1.98–2.99**
High: 3–5	2.8	1313	3.0	1377	6.6	44	2.9	2734	**0.91**	**0.84–0.98**	**2.06**	**1.50–2.83**	**2.27**	**1.66–3.11**
Very high: 6–12	0.8	401	1.1	495	3.5	23	1.0	23	1.02	0.89–1.17	**3.24**	**2.11–5.00**	**3.18**	**2.07–4.88**
Hyperactive/Distraction														
Low: 0–8	61.7	29,474	78.1	36,183	77.5	516	69.8	66,173	Ref		Ref		Ref	
Moderate: 9–10	10.1	4835	7.6	3528	9.9	66	8.9	8429	**0.66**	**0.63–0.69**	0.99	0.77–1.29	**1.51**	**1.16–1.89**
High: 11–12	11.3	5379	6.5	3016	7.2	48	8.9	8443	**0.55**	**0.52–0.57**	0.79	0.59–1.06	**1.45**	**1.07–1.95**
Very high: 13—16	17.0	8116	7.8	3606	5.4	36	12.4	11,758	**0.47**	**0.45–0.49**	**0.51**	**0.36–0.71**	1.08	0.77–1.52
Social disengagement scale														
Low: 0–2	70.3	32,679	61.6	27,495	45.1	279	65.9	60,453	Ref		Ref		Ref	
Moderate: 3–5	16.8	7825	19.7	8801	24.6	152	18.3	16,778	**1.05**	**1.01–1.09**	**1.38**	**1.13–1.69**	**1.32**	**1.08–1.61**
High: 6–8	5.6	2620	7.8	3464	10.5	65	6.7	6149	**1.16**	**1.09–1.22**	**1.57**	**1.19–2.06**	**1.36**	**1.03–1.78**
Very high: 9–16	7.2	3368	10.9	4877	19.9	123	9.1	8368	**1.17**	**1.11–1.23**	**1.99**	**1.60–2.49**	**1.71**	**1.37–2.13**

% and *N* indicate percent of gender and number of individuals, respectively. Odds ratios adjusted for age and 95% confidence intervals are indicated by OR and 95% CI, respectively. The reference for each odds ratio is indicated as ref. Significant adjusted odds ratios are bolded

Except for positive symptoms, cisgender males and females showed significant differences in all measures. Compared to cisgender males and adjusting for age, cisgender female cases were more likely to be at risk of suicide, experienced physical, sexual, emotional abuse, bullying, exhibited more depressive or anxiety symptoms, and were more socially disengaged. Cisgender female cases were less likely to be recorded with the following: a strong and supportive family relationship, family member(s) feeling overwhelmed by the youth’s condition, being at high risk of causing injury to others, having externalizing mental health problems, or being hyperactive or distractible.

When comparing those with TGNC gender to cisgender males, similar and more pronounced patterns were observed when comparing cisgender females to cisgender males. In addition, positive symptoms were significantly more likely for TGNC gender compared to the other two groups.

Comparing TGNC gender to cisgender females, differences either diminished, or for some measures, disappear entirely, compared to TGNC versus cisgender males. For example, no differences between TGNC and cisgender females were found with respect to risk of injury to others, physical assault, sexual assault, and bullying. Externalizing problems showed only minor differences between TGNC and cisgender females.

## Discussion

The current study adds to the growing body of literature on TGNC populations by investigating the mental health presentations of treatment-seeking TGNC, cisgender male, and cisgender female youth in Ontario, Canada. Cisgender males and females presented with significant mental health differences on all measures except positive symptoms. Cisgender males and TGNC individuals also exhibited many significant differences in their mental health presentations while cisgender females and TGNC individuals exhibited the fewest significant differences. In fact, the mental health presentations of TGNC youth resembled their cisgender female peers in many ways, and this was likely related to the fact that the majority of these cases were recorded as biologically female. This suggests that there may be a biological component underlying these similar mental health presentations across both identities, as previously suggested by Eisenberg and colleagues [1].

With respect to accessing mental health services, cisgender males presented at the youngest age, with treatment-seeking cisgender males being on average 1.7 years younger than cisgender females. Cisgender males were more likely to demonstrate a risk of harm to others, externalizing behaviours, and hyperactivity and distractibility than cisgender females and TGNC youth. These results are consistent with other extant literature which demonstrates that males, when compared to females, have a higher prevalence of externalizing disorders [32, 33], hyperactivity [34, 35], conduct disorders, and aggression [32].

On the other hand, cisgender females showed higher adjusted odds of depression, anxiety, risk of suicide and self-harm, and social disengagement than cisgender males. Cisgender females also had 62% higher adjusted odds of reporting a self-injurious attempt to kill themselves than their cisgender male counterparts. In the past year, cisgender females had 448% higher adjusted odds of being a victim of sexual assault/abuse than cisgender males. Findings reported herein are consistent with the literature examining gender differences demonstrating that females have higher rates of mood and anxiety disorders [33, 36], self-harm [37–39], childhood sexual abuse [40], and are at a higher risk of attempting suicide [41] when compared to males.

Based on our findings, TGNC youth exhibited mental health needs that were consistent with older cisgender females with higher acuity levels. In line with previously cited literature, these individuals showed significantly heightened mental health concerns compared to both cisgender males and females including anxiety, depression, social disengagement, self-harm, and risk of suicide. For example, TGNC youth had 932% higher adjusted odds of being very high risk for suicide and self-harm behaviours than cisgender males, and 355% higher adjusted odds compared to cisgender females. Moreover, TGNC youth were found to have 196% higher adjusted odds of reporting a self-injurious attempt to kill themselves compared to cisgender males and 82% higher adjusted odds compared to cisgender females. These findings contribute to the literature demonstrating significant mental health disparities regarding a heightened risk for suicide and self-harm in this population [1, 3, 6, 10–13, 16, 17].

Levels of victimization within the past year, such as bullying, sexual, and physical abuse, were not significantly different among cisgender females and TGNC youth, with the exception of emotional abuse. Though previous literature indicates that TGNC youth are at increased risk of abuse [3–5], bullying, and victimization [1, 5–8], our results indicate minimal differences. It should be noted that since TGNC youth were the oldest age group, on average, and the present study only examined abuse within the past year, perhaps lifetime prevalence of this abuse would demonstrate increased levels of victimization for this population. Given that these are treatment-seeking youth, differences among groups may also be non-significant due to the fact that approximately 50% of youth seeking mental health services have experienced some type of trauma or abuse.

Positive symptoms were significantly more likely to be present in the TGNC population when compared to cisgender males and females. For example, TGNC individuals were found to have 224% higher adjusted odds of showing very high levels of positive symptoms than cisgender males and 218% higher adjusted odds compared to cisgender females. One potential explanation for this is that adverse childhood experiences and trauma, such as abuse, bullying, and victimization, have been associated with an increased risk of psychosis [42, 43]. Past literature suggests that TGNC populations are exposed to many adverse childhood experiences and traumas [1, 3–8]. Other research has indicated that certain biological factors (e.g., estrogen and related sex hormones) may also play a significant role in the development of positive symptoms given that estrogen has been found to be a protective factor in the development of psychosis [44, 45]. However, future research is needed to further explore the association between positive symptoms and TGNC youth, as this has been largely unexplored in the literature to date, and may vary depending on the samples utilized in these research studies (e.g., comparison of community versus clinically-referred samples).

Overall, the family relationships were noted to be less supportive and strong with TGNC youth compared to the other groups. Such findings are consistent with previous literature reporting a perceived lack of social support in this population [1, 3]. Lower social support has been associated with negative mental health symptomology in gender and sexual minority individuals [10, 12, 46–50], which may explain why TGNC youth are experiencing heightened levels of mental health symptoms. Furthermore, TGNC populations experience disproportionate levels of discrimination, bias, stigma, harassment, lack of acceptance, and other societal stressors which negatively impact mental health [50–53]. Given the importance of the role of protective factors, such as family and community connectedness and social support, in buffering against the adverse mental health outcomes in TGNC youth [50, 52, 54–58], it is imperative that programs, policies, and resources are available for youth in these communities.

### Limitations and Future Directions

While this study has many strengths, it is not without limitations. First, all possible TGNC subgroups were not explored and, as such, the mental health outcomes of the many possible identities within this population. These identities which exist within the broader TGNC framework are associated with varying degrees of mental health outcomes in the literature. For example, TGNC individuals who have the additional identification of a sexual minority have significantly higher levels of anxiety and depression compared to those who identify with only one minority identity, indicating that multiple minority identities may have an additive effect on mental health [14]. Therefore, without further demographic knowledge of our sample, it cannot be determined whether the results are being driven by a particular identity within the TGNC population. Rather, the results are interpreted as pertaining the TGNC experience as a whole. Future research should examine the associations with mental health and the various identities within the TGNC population to ensure that those with heightened risk for mental health difficulties are accurately represented in the literature.

Furthermore, the data used in the present study classifies sex and gender into three categories: male, female, and other. Unfortunately, since the individuals in the sample were not explicitly asked about their gender identity, the present study assumes that the category of other represents TGNC individuals, and male and female represents cisgender individuals. Thus, we cannot be certain that the category of “other” captures all individuals who identify as TGNC. For example, there are a number of individuals in the sample who may be transgender but did not identify their gender as “other”, preferring instead to identify their gender as male or female. Therefore, there is a possibility that our sample may not only underrepresent the TGNG population as a whole, but also specifically underrepresent TGNC individuals who identify with one of the binary options of male or female. TGNC individuals whose gender identity is male or female may experience less mental health risks, such as lower levels of anxiety and depression, than TGNC individuals who identify as non-binary [59, 60], and therefore the mental health presentations of TGNC youth in the present study may be heightened when compared to other TGNC populations.

It should also be noted that the present study did not examine a variety of factors which may have an impact on the results, such as psychotropic medications, maternal depression, and parental aggression. All of these factors have the potential to alter both the brain and behaviour [61–65]. Therefore, these factors could potentially be influencing the results found in the present study and future studies should consider these factors in their analyses.

### Clinical Implications

The present study adds to the growing body of literature investigating the unique mental health presentations of TGNC youth. On average, TGNC youth displayed heightened mental health concerns including anxiety, depression, positive symptoms, experiences of emotional abuse, and risk of suicide and self-harm when compared to cisgender males and females. As a result, there is a critical need for clinicians to provide gender-affirming and competent care in order to meet the mental health needs of these youth. This begins with a personal examination of the clinician’s own biases or assumptions, as well as appropriate education and training to increase their understanding and ability to empathize with TGNC youth’s experiences [66–68]. TGNC clients report taking on the role of educating their clinician on the TGNC experience, also known as education burdening, as a common barrier to care [66, 68]. Clinicians must also appreciate the diverse narratives of TGNC people and acknowledge each individual’s unique experience to avoid underemphasizing, generalizing, pathologizing, or stigmatizing [68].

Given the discrimination, stigmatization and prejudice experienced by TGNC youth, concerns about being accepted by clinicians and other medical professionals is another common barrier to care [66, 67, 69, 70]. Clinicians can combat this barrier by being connected with organizations which have already built trust with the TGNC community, as well as by demonstrating inclusion through creating a gender-affirming and welcoming healthcare setting [71].

Furthermore, due to the lack of social support and stigma experienced by this population, clinicians should focus on increasing awareness about and emphasizing client’s engagement in protective factors which facilitate resilience, such as support groups and TGNC communities [67]. On a broader societal level, efforts to create social change through policy and advocacy can further aid in creating a culture that is affirming of TGNC youth [71]. By improving societal acceptance, social support, and community connectedness, clinicians and society as a whole can aid in buffering against the negative effects of discrimination, stigmatization, and prejudice on TGNC individual’s mental health.

## Summary

Further understanding of the mental health needs of TGNC youth is needed to ensure clinicians provide competent and accurately informed care-planning for this population. The present study compares the mental health presentations of the TGNG, and cisgender male and female youth by examining 94,804 youth seeking treatment at mental health service agencies across the Province of Ontario. At intake, data from the ChYMH or ChYMH-S was collected through trained assessors as part of standard of care. Analyses of the data was conducted to reveal significant differences in mental health presentations across TGNC and cisgender male and female identities. Cisgender males presented in the mental health system at the youngest age and showed significant differences on all measures, except positive symptoms, when compared to cisgender females. Controversially, TGNC youth presented in the mental health system at the oldest age and showed many similarities in mental health needs to cisgender females, and had even more pronounced differences when compared to the cisgender males. Despite many similarities to cisgender females, TGNC youth showed a significantly more pronounced risk for suicide/self-harm, symptoms of anxiety and depression, social disengagement, positive symptoms, past suicide attempts, being a victim of emotional abuse, and not having a strong supportive relationship with their family. In summary, compared to cisgender males and females, TGNC youth were more likely to report many mental health concerns, notably including life-threatening ones, in addition to being less likely to have familial supports. Understanding these unique mental health presentations is critical for clinicians educating themselves on the experiences of TGNC youth in order to best inform care-planning. An emphasis on fostering supportive communities and reducing societal stigma is a critical step forward in providing safe spaces for TGNC youth to seek support and mental health services to aid in mitigating negative mental health outcomes.
